# Construction and validation of a predictive model for postoperative stent occlusion in patients undergoing iliac vein stenting based on an explainable machine learning model

**DOI:** 10.3389/fsurg.2025.1707615

**Published:** 2025-11-18

**Authors:** Yan Jiang, Dan Nie, Lifeng Zhang, Xia Tang, Haolu Li, Long Li, Huqiang He, Yang Liu, Weijian Mao, Zhiwei Xiong, Chengyong Jin

**Affiliations:** 1 Department of Vascular Surgery, Ya'an Hospital of Traditional Chinese Medicine, Ya'an, Sichuan, China; 2 Emergency Department, General Hospital of Western Theater Command, PLA, Chengdu, Sichuan, China; 3 Department of Emergency and Critical Care Medicine, The 945th Hospital of the Joint Logistics Support Force, PLA, Ya’an, Sichuan, China; 4 Department of Vascular Surgery, Affiliated Hospital of Southwest Medical University, Luzhou, Sichuan, China; 5 Department of Vascular Surgery, Affiliated Hospital of Chengdu University of Traditional Chinese Medicine, Chengdu, Sichuan, China; 6 Department of Vascular Surgery, Zigong Fourth People's Hospital, Zigong, Sichuan, China; 7 Department of Vascular Surgery, Panzhihua Central Hospital, Panzhihua, Sichuan, China

**Keywords:** iliac venous stent occlusion, explainable machine learning, automated machine learning (AutoML), risk prediction, clinical decision support system

## Abstract

**Objective:**

This study aims to develop an interpretable machine learning model for predicting post-operative iliac venous stent occlusion risk.

**Methods:**

Employing a retrospective cohort design, data from 826 patients across seven hospitals (January 2017–June 2024) were incorporated with stratified sampling into training (*n* = 661) and test sets (*n* = 165), ensuring no significant baseline characteristic differences (all *P* > 0.05). An AutoML framework was constructed using the Improved Sequoia Optimization Algorithm (ISequoiaOA), integrated with LASSO feature selection and SHAP interpretability analysis; model evaluation incorporated six core metrics (including AUC/PR-AUC), calibration performance, and Decision Curve Analysis (DCA).

**Results:**

In independent testing-set validation, the AutoML model demonstrated superior robustness: ROC-AUC reached 0.9251 and PR-AUC 0.8712. Decision curve analysis confirmed significantly higher clinical net benefit across a wide threshold probability range (1%–87%) compared to conventional approaches, indicating exceptional generalizability. Calibration curves revealed the lowest Brier score (0.123) in the test set, further validating predictive accuracy. Outperforming comparative models [e.g., XGBoost [ROC-AUC 0.8203] and LightGBM [PR-AUC 0.7806]], AutoML dominated across all metrics including accuracy (0.7417) and F1-score (0.7559). Concurrently, SHAP analysis quantified critical feature contributions: Pathogenic triad (DVT + Cockett + PE); Hemodynamic thresholds (common femoral and external iliac vein recanalization rates both <70%); Stent geometric parameters (diameter >14 mm/inferior vena cava segment length >20 mm); With CRP > 10 mg/L and D-dimer > 1.5 mg/L coexistence elevating occlusion risk.

**Conclusion:**

The occlusion prediction system integrating AutoML with explainable AI successfully quantifies multi-level interactions, surpassing traditional predictive dimensions to provide evidence-based support for personalized anticoagulation and stent optimization.

## 1 Introduction

Iliac vein stenting, as a core interventional approach for treating iliac vein compression syndrome, post-thrombotic syndrome, and iliac vein obstructive diseases, has been widely promoted in clinical practice due to its advantages of minimal invasiveness and rapid recovery, becoming an effective method to improve hemodynamic disorders in the lower limbs of patients (1, 2). However, in-stent restenosis after stent implantation is a frequently occurring severe complication, with persistently high incidence rates, forming a key bottleneck that constrains therapeutic efficacy improvement (3, 4). This complication not only leads to symptom recurrence and increased rehospitalization rates but also may trigger catastrophic consequences such as fatal pulmonary embolism, severely affecting patients' long-term prognosis and quality of life (5). The underlying pathological mechanisms are complex and multifactorial, involving interactions among multiple layers of factors, such as incomplete venous outflow tract recanalization, abnormal vascular wall inflammatory states, persistent hypercoagulable conditions, specific anatomical risks, and dynamic imbalances in the coagulation-anticoagulation system (6–8). This issue is particularly prominent in specific high-risk populations, such as patients with deep vein thrombosis (DVT) combined with Cockett syndrome and pulmonary embolism (PE), where the occlusion risk often increases exponentially.

In current clinical practice, the prediction of stent occlusion risk faces significant limitations, primarily characterized by strong experiential reliance and decision-making processes excessively dependent on single risk factors or physicians' individual experience, while neglecting the synergistic effects and non-linear interactions among multi-dimensional characteristics. For instance, prediction based solely on one-dimensional indicators such as D-dimer levels fails to capture the amplification effect of thrombus formation when inflammatory factors coexist with a hypercoagulable state (2, 4). Furthermore, traditional risk scores predominantly focus on demographic or routine laboratory metrics, lacking systematic integration of post-operative hemodynamic recovery indicators, stent design parameters, and specific pathological conditions (9). Existing models are mostly constructed using static linear regression, which struggles to handle non-linear relationships and threshold effects among complex clinical features. This deficiency in predictive dimensionality results in models lacking calibration performance and clinical net benefit in practical applications, restricting their value in anticoagulation strategy optimization or stent parameter adjustment; innovative methods are urgently needed to overcome this translational bottleneck (10).

In addressing such highly non-linear, multi-dimensional medical prediction problems, machine learning (Machine Learning, ML) demonstrates immense potential. As a core branch of artificial intelligence, ML overcomes the limitations of traditional statistical models by leveraging algorithms to mine hidden patterns and associations from large-sample, high-noise clinical data (11, 12). Algorithms such as support vector machines (SVM) and gradient boosting decision trees have shown significant advantages in processing non-linear relationships, enabling automatic feature selection, and enhancing prediction accuracy (13). However, the broad application of ML in healthcare still faces two major challenges: the “black box” dilemma and automation difficulties (14, 15). Complex models like deep learning deliver excellent predictive power, but their internal decision logic lacks transparency, undermining physician trust and hindering clinical adoption; resolving this issue requires the use of explainability analysis tools, such as feature importance rankings and intervention effect visualization, to enhance model transparency (16). Simultaneously, conventional ML modeling processes are cumbersome, relying on manual feature engineering and hyperparameter tuning, resulting in high barriers to model construction and insufficient generalization (17, 18). The proposal of automated machine learning (Automated Machine Learning, AutoML) frameworks aims to reduce development complexity through end-to-end automation, improving model reproducibility and application efficiency across different datasets, but their practicality and robustness in complex clinical scenarios still need validation (19).

To address these challenges, this study aims to construct a predictive model for postoperative stent occlusion in iliac vein stenting that integrates high precision, strong robustness, and good interpretability, thereby providing intelligent decision support for clinical personalized risk management. Our core objectives focus on three aspects: First, by effectively integrating patient baseline characteristics, underlying etiology profiles, key surgical design parameters, and postoperative dynamic monitoring indicators, we establish a multidimensional dataset that comprehensively captures thrombosis risk factors to systematically cover multi-layered predictors. Second, emphasizing the unification of predictive model performance and interpretability, we explore the use of advanced AutoML technology for end-to-end model construction; this enhances discriminative performance metrics such as the area under the ROC curve while deeply integrating feature selection with explainable artificial intelligence techniques to reveal combinatorial interaction rules and threshold effects of key predictors, ensuring model logic is transparent and interpretable. Finally, going beyond mere accuracy metrics, we introduce calibration curves and decision curve analysis (DCA) to rigorously evaluate model probability output accuracy and clinical net benefit, clarifying practical threshold intervals and application value for real-world diagnosis and treatment. This research not only applies cutting-edge ML techniques to solve the complex problem of iliac vein stent occlusion prediction but also commits to developing practical tools to provide an evidence-based foundation for patient stratification and refined treatment, thereby promoting long-term vascular patency and quality-of-life improvement.

## 2 Methods

### 2.1 Study population

This study employed a retrospective cohort design, selecting patients who underwent iliac vein stenting between January 2017 and June 2024 across seven public hospitals as the study subjects. After applying inclusion and exclusion criteria, the final study population comprised 826 cases. Since it was a retrospective study, patient informed consent was exempted; the study was approved by the Ethics Committee of the principal research unit, Ya'an Hospital of Traditional Chinese Medicine (Ethics Approval No.: 202508), and conducted in accordance with the relevant standards and requirements of the World Medical Association's Declaration of Helsinki.

Inclusion Criteria: (1) Underwent iliac vein stenting surgery; (2) Iliac vein stent occlusion or patency was confirmed during the follow-up period using color Doppler ultrasonography, computed tomography venography (CTV), or digital subtraction angiography (DSA); (3) Complete medical records.

Exclusion Criteria: (1) Lost-to-follow-up cases; (2) Patients with malignant tumors and life expectancy of less than three months.

### 2.2 Data collection

All patient data were sourced from hospital electronic medical record systems and extracted in a structured manner, with verification performed by two certified researchers. Data types primarily encompassed the following aspects: (1) General Information: Gender, age, body mass index (BMI), hospitalization length in days, etiology classification (PTS/DVT/Cockett/DVT + Cockett/DVT + Cockett + PE), smoking history (yes/no), presence of arteriovenous fistula (yes/no); (2) Perioperative Indicators: Surgery duration in minutes, number of stents, total stent length in mm, stent diameter in mm, stent protrusion length into the inferior vena cava in mm; postoperative anticoagulation regimen [warfarin/new oral anticoagulant (NOAC)/no anticoagulation], compression therapy (yes/no); postoperative initial coagulation indicators: prothrombin time (PT) in s, activated partial thromboplastin time (APTT) in s, fibrinogen (FIB) in g/L, D-dimer in mg/L FEU, C-reactive protein (CRP) in mg/L; (3) Postoperative Recovery Indicators: Recanalization rates (%) at the first ultrasound review (30 days post-surgery) for the external iliac vein, common femoral vein, and femoral vein. The primary outcome measure was stent occlusion occurring within one year after stenting, confirmed by vascular ultrasound.

### 2.3 Model establishment

**Figure 1:**
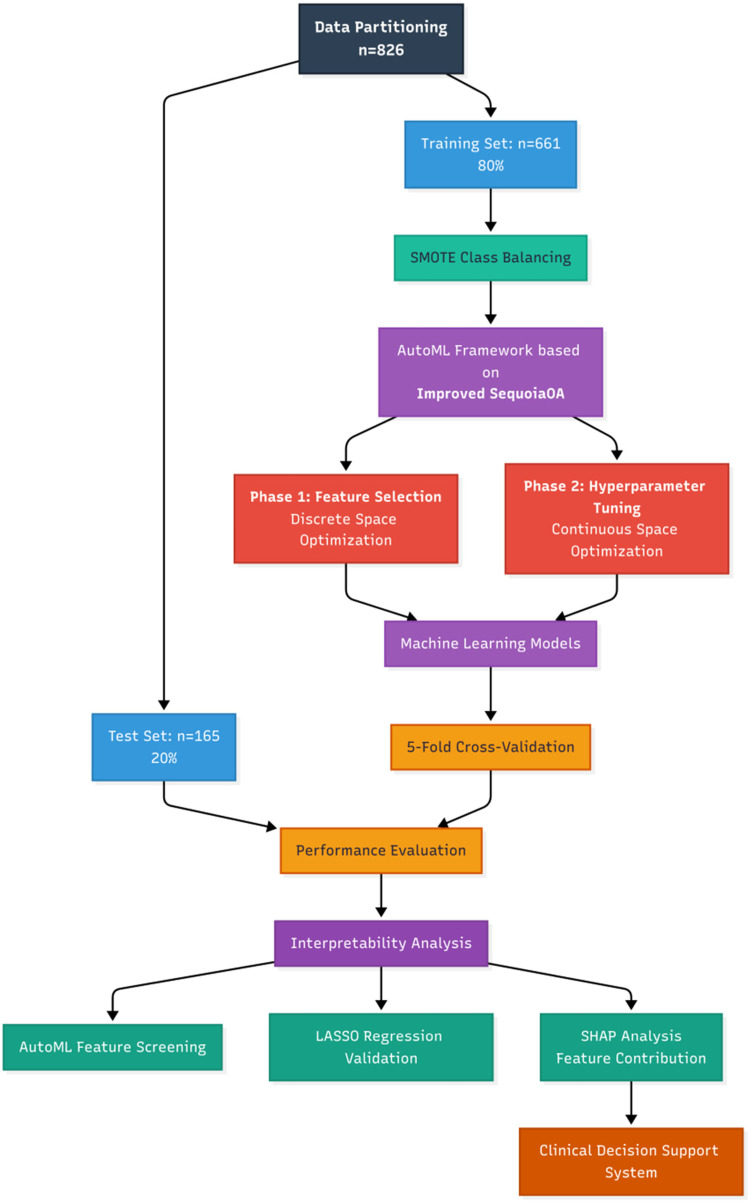
Research flowchart.

Based on the outcome indicators, the study cohort was randomly stratified into training and testing sets at an 8:2 ratio (training set: *n* = 661; testing set: *n* = 165). This study proposes an adaptive machine learning framework based on the Improved Sequoia Optimization Algorithm (ISequoiaOA), termed Automated Machine Learning (AutoML), aimed at simultaneously achieving key feature selection and hyperparameter optimization for postoperative stent occlusion prediction in iliac vein stenting. The Sequoia Optimization Algorithm (SequoiaOA) is a novel metaheuristic algorithm (intelligent optimization algorithm), inspired by the self-regulating dynamics and resilience derived from the Sequoia forest ecosystem. SequoiaOA was refined into ISequoiaOA using chaotic mapping to optimize the initial population distribution for enhanced spatial exploration, and a dynamic Lévy flight step control strategy to balance search efficiency in the exploration and exploitation phases. To further validate the performance of ISequoiaOA, it was assessed using the CEC2022 benchmark test function performance. This framework employs a dual-stage collaborative optimization process: the first stage involves screening high-weight feature subsets in a discrete space, while the second stage refines hyperparameter tuning in the continuous space. Concurrently, six comparative models were established: Logistic Regression (LR), Support Vector Machine (SVM), Adaptive Boosting (AdaBoost), Extreme Gradient Boosting (XGBoost), Light Gradient Boosting Machine (LightGBM), and the proposed AutoML framework. All models were implemented on the MATLAB 2024b platform, with data standardized during preprocessing and evaluated using five-fold cross-validation; Synthetic Minority Oversampling Technique (SMOTE) was applied to address class imbalance issues. The specific research process is shown in Figure 1.

### 2.4 Evaluation metrics

This study established a multidimensional evaluation system comprising: (1) Classification performance: For the predictive model, six core metrics were adopted to systematically assess discriminative ability and stability in class-imbalanced scenarios: Accuracy (ACC), Sensitivity (SEN), Specificity (SPE), Precision (PRE), F1 score (harmonic mean of precision and recall), Area Under the ROC Curve (ROC-AUC), and Area Under the Precision-Recall Curve (PR-AUC). (2) Calibration performance: Calibration curves combined with the Brier score (lower scores indicate higher prediction accuracy) were used to evaluate probability prediction precision. (3) Clinical application: Decision Curve Analysis (DCA) was applied to quantify clinical utility by calculating the net benefit (NB) at different threshold probabilities:

$$NB=\frac{TP}{N}-\frac{FP}{N}\times \frac{\;p_{t}}{1-p_{t}}$$

where *TP* is true positives, *FP* is false positives, *N* is total sample size, and *pt* is the risk threshold. By comparing *NB* with reference lines for traditional intervention strategies, the effective interval for model-assisted decision-making was validated.

### 2.5 Interpretability analysis

After initial screening of prognostic prediction features via the AutoML framework, Lasso regression analysis was applied to verify feature robustness, followed by SHAP interpretability modeling to analyze clinical rationality. The specific workflow encompassed: (1) AutoML feature preliminary screening: Based on predefined search spaces and optimization objectives, the AutoML algorithm automatically identified feature subsets significantly associated with prognosis; (2) Lasso feature validation: Applied to the feature subset screened by AutoML, Lasso regression validated sparsity and stability through regularization constraints, ensuring anti-overfitting capability of key features, while differences between Lasso and AutoML-selected features were compared; (3) SHAP (Shapley Additive Explanations) interpretability analysis: Based on game theory, the SHAP algorithm quantified feature contributions, revealing the overall influence intensity of key variables through global feature importance ranking and enabling visualization of prediction logic to verify rationality.

### 2.6 Clinical decision system

In our study, MATLAB's App Designer function was utilized to develop a clinical decision support software. This software integrates the constructed predictive model to provide clinicians with an intuitive, user-friendly tool for prognosis assessment in patients. The software is web-deployable for convenient clinical use.

### 2.7 Statistical methods

All study data were uniformly imported into the SPSS 26.0 statistical analysis platform for standardized processing. Continuous variables with normal distribution were expressed as mean ± standard deviation $\left(\bar{x}\pm s\right)$, while those with non-normal distribution were denoted as median (interquartile range) [M (IQR)]; categorical variables were expressed as frequency and percentage [*n* (%)]. For inter-group comparisons, continuous variables first underwent normality testing: if both groups conformed to normal distribution, univariate *t*-test was applied; otherwise, Mann–Whitney *U* test was used. Categorical variables were compared using Pearson's chi-square test. Statistical significance was determined based on *p*-values (two-tailed test, significance threshold *α* = 0.05), and results were presented in tabular formats.

## 3 Results

### 3.1 Baseline data of study population

A total of 826 patients were included in our study, of whom 128 (15.5%) experienced stent occlusion, while 698 (84.5%) did not. No significant statistical differences were observed between the training set (*n* = 661) and test set (*n* = 165) across all baseline characteristics and laboratory indicators (all *P* > 0.05). The stratified random sampling was effective: the proportion of postoperative stent occlusion was highly consistent between the two groups (training set 15.58% vs. test set 15.15%; *χ*^2^ = 0.024, *P* = 0.877). For details, see Table 1.

**Table 1:** Comparison of clinical characteristics between training set and testing set.

Feature	Training set (*n* = 661)	Test set (*n* = 165)	Statistic	*P*-value
Outcome				
Stent occlusion, *n* (%)	103 (15.58%)	25 (15.15%)	*χ*^2^ = 0.019	0.891
Demographics				
Male, *n* (%)	396 (59.91%)	101 (61.21%)	*χ*^2^ = 0.094	0.760
Age (years), Mean ± SD	58.92 ± 12.43	57.86 ± 13.07	*t* = 0.970	0.332
BMI (kg/m^2^), Median [IQR]	25.8 [23.1–28.5]	25.6 [22.8–28.7]	*U* = 53,142	0.653
Hospital stay (days), Median [IQR]	7.0 [5.0–10.0]	7.0 [5.0–11.0]	*U* = 54,201	0.482
Etiology, *n* (%)			*χ*^2^ = 1.624	0.804
PTS	215 (32.53%)	52 (31.52%)	–	–
DVT	178 (26.93%)	48 (29.09%)	–	–
Cockett	102 (15.43%)	23 (13.94%)	–	–
DVT + Cockett	121 (18.31%)	34 (20.61%)	–	–
DVT + Cockett + PE	45 (6.81%)	8 (4.85%)	–	–
Smoking history, *n* (%)	227 (34.34%)	60 (36.36%)	*χ*^2^ = 0.238	0.626
Arteriovenous fistula, *n* (%)	51 (7.72%)	12 (7.27%)	*χ*^2^ = 0.037	0.848
Perioperative				
Surgery time (min), Mean ± SD	86.51 ± 24.36	84.29 ± 25.73	*t* = 1.035	0.301
Stent number, Median [IQR]	1.0 [1.0–2.0]	1.0 [1.0–2.0]	*U* = 53,076	0.618
Total stent length (mm), Mean ± SD	98.73 ± 25.52	101.24 ± 35.18	*t* = 1.041	0.298
Stent diameter (mm), Mean ± SD	14.29 ± 2.17	14.03 ± 2.32	*t* = 1.358	0.175
IVC extension (mm), Median [IQR]	20.0 [15.0–25.0]	20.0 [15.0–26.0]	*U* = 54,128	0.507
Anticoagulation, *n* (%)			*χ*^2^ = 0.910	0.635
Warfarin	312 (47.20%)	75 (45.45%)	–	–
NOAC	298 (45.08%)	80 (48.48%)	–	–
None	51 (7.72%)	10 (6.06%)	–	–
Compression therapy, *n* (%)	508 (76.85%)	130 (78.79%)	*χ*^2^ = 0.281	0.596
PT (s), Mean ± SD	12.83 ± 1.32	12.97 ± 1.42	*t* = 1.200	0.230
APTT (s), Mean ± SD	32.56 ± 4.23	32.71 ± 4.43	*t* = 0.404	0.687
FIB (g/L), Median [IQR]	4.05 [3.32–4.89]	4.12 [3.40–4.95]	*U* = 52,739	0.692
D-dimer (mg/L), Median [IQR]	1.25 [0.78–1.96]	1.30 [0.81–2.05]	*U* = 52,048	0.433
CRP (mg/L), Median [IQR]	8.6 [4.2–14.3]	9.1 [4.5–15.0]	*U* = 51,876	0.384
Postoperative recovery				
EIV recanalization (%), Mean ± SD	68.37 ± 18.53	67.16 ± 19.24	*t* = 0.745	0.457
CFV recanalization (%), Median[IQR]	75.0 [62.0–87.0]	74.0 [60.0–86.0]	*U* = 53,827	0.564
FV recanalization (%), Median [IQR]	70.0 [58.0–82.0]	72.0 [58.3–83.0]	*U* = 53,645	0.602

### 3.2 Algorithm performance improvement test

**Figure 2:**
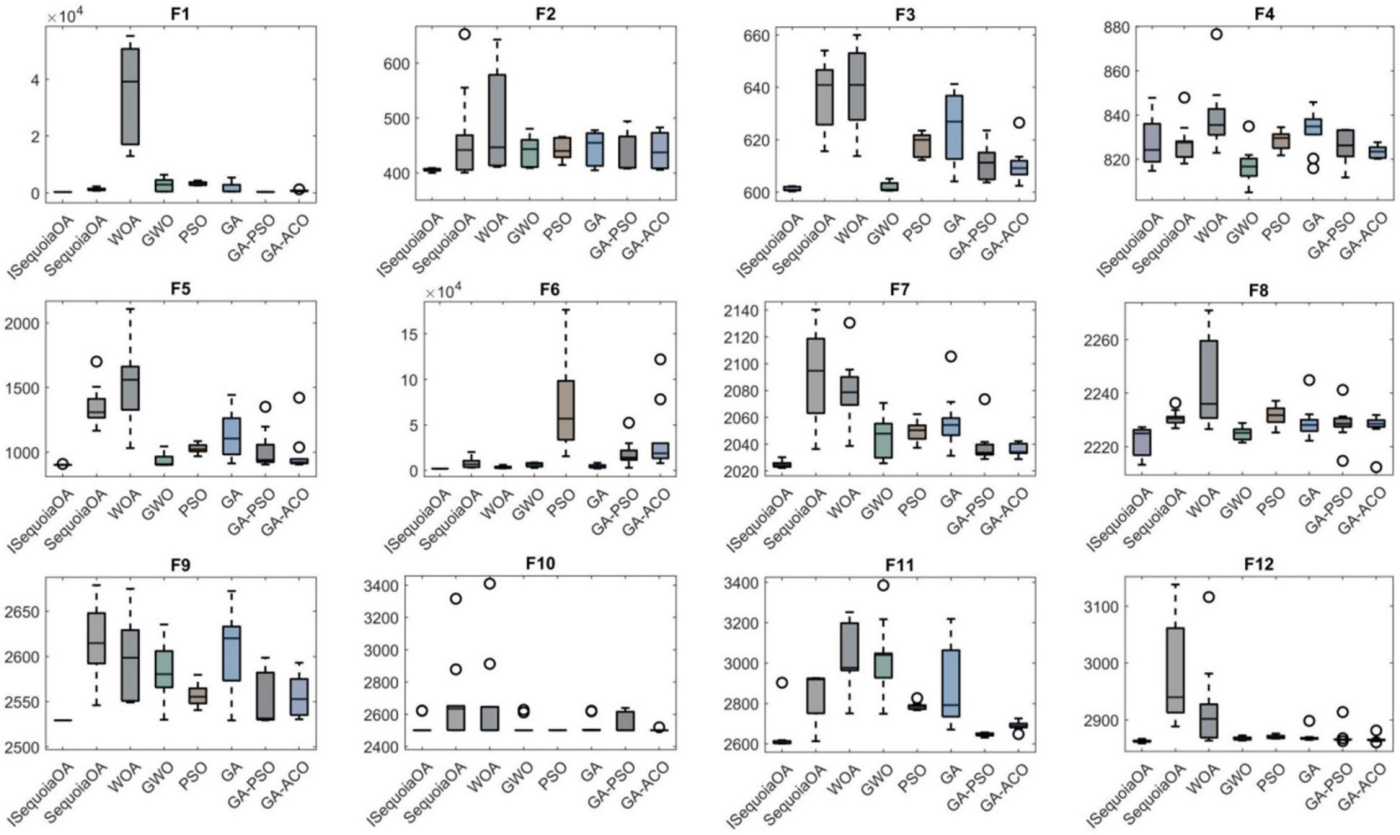
Comparative performance of swarm intelligence algorithms.

**Figure 3:**
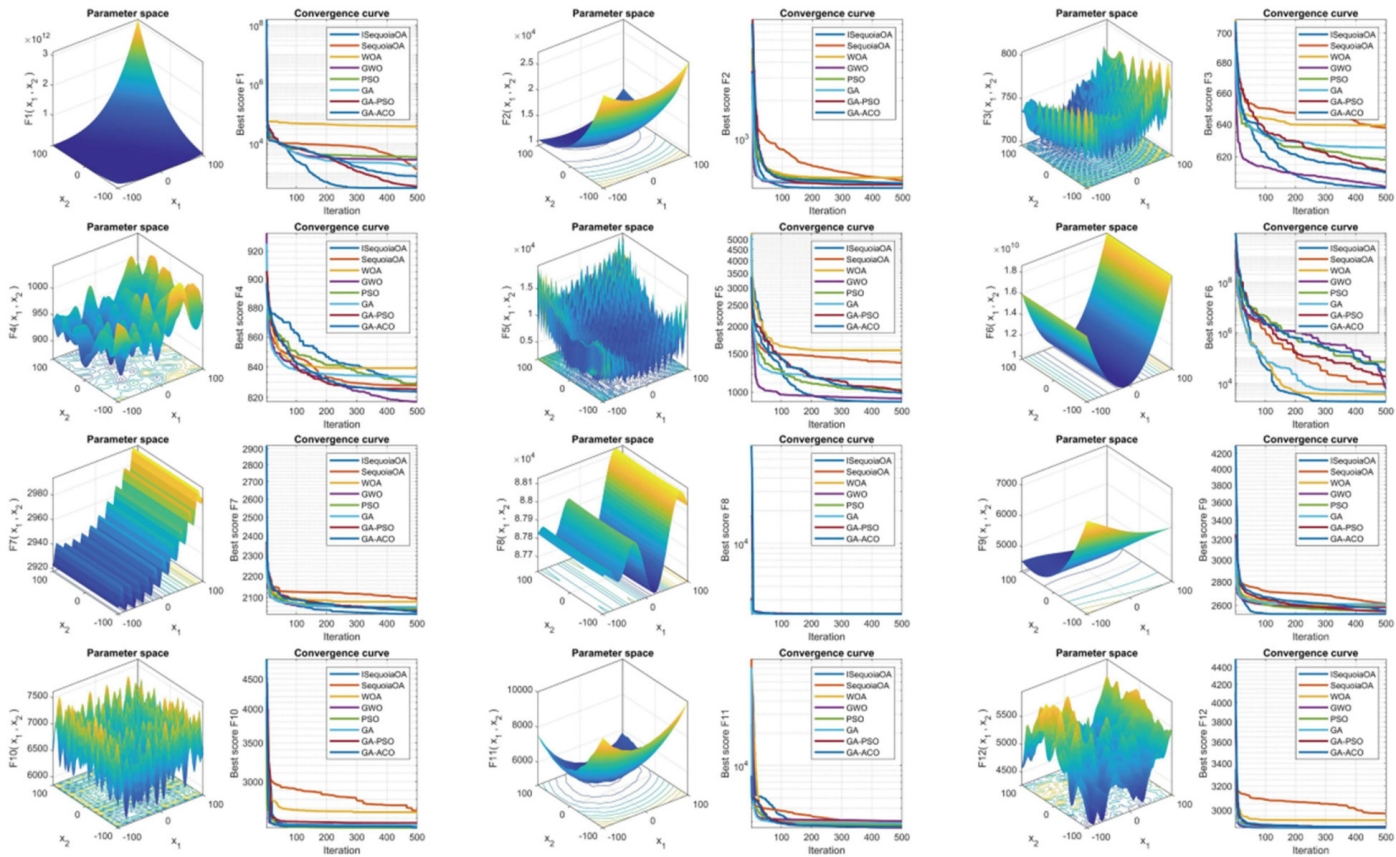
Convergence performance of swarm intelligence algorithms.

To verify the optimization capabilities of the improved ISequoiaOA algorithm, its performance was compared with the original SequoiaOA, WOA, GWO, PSO, GA, GA-PSO, and GA-ACO algorithms. Experiments used all 12 benchmark functions from the CEC2022 test set, with variable dimensions set to 10, population size to 30, maximum iterations to 500, and 30 independent runs to ensure statistical reliability. Box plots were generated to evaluate optimization stability based on the 30 results, revealing that ISequoiaOA outperformed in most test functions, significantly surpassing the original SequoiaOA and other algorithms in stability (Figure 2). Further convergence curve analysis demonstrated that ISequoiaOA achieved faster convergence with minimal risk of local optima during iterations (Figure 3). These results confirm ISequoiaOA's superior global optimization and convergence efficiency.

### 3.3 Model training results

**Figure 4:**
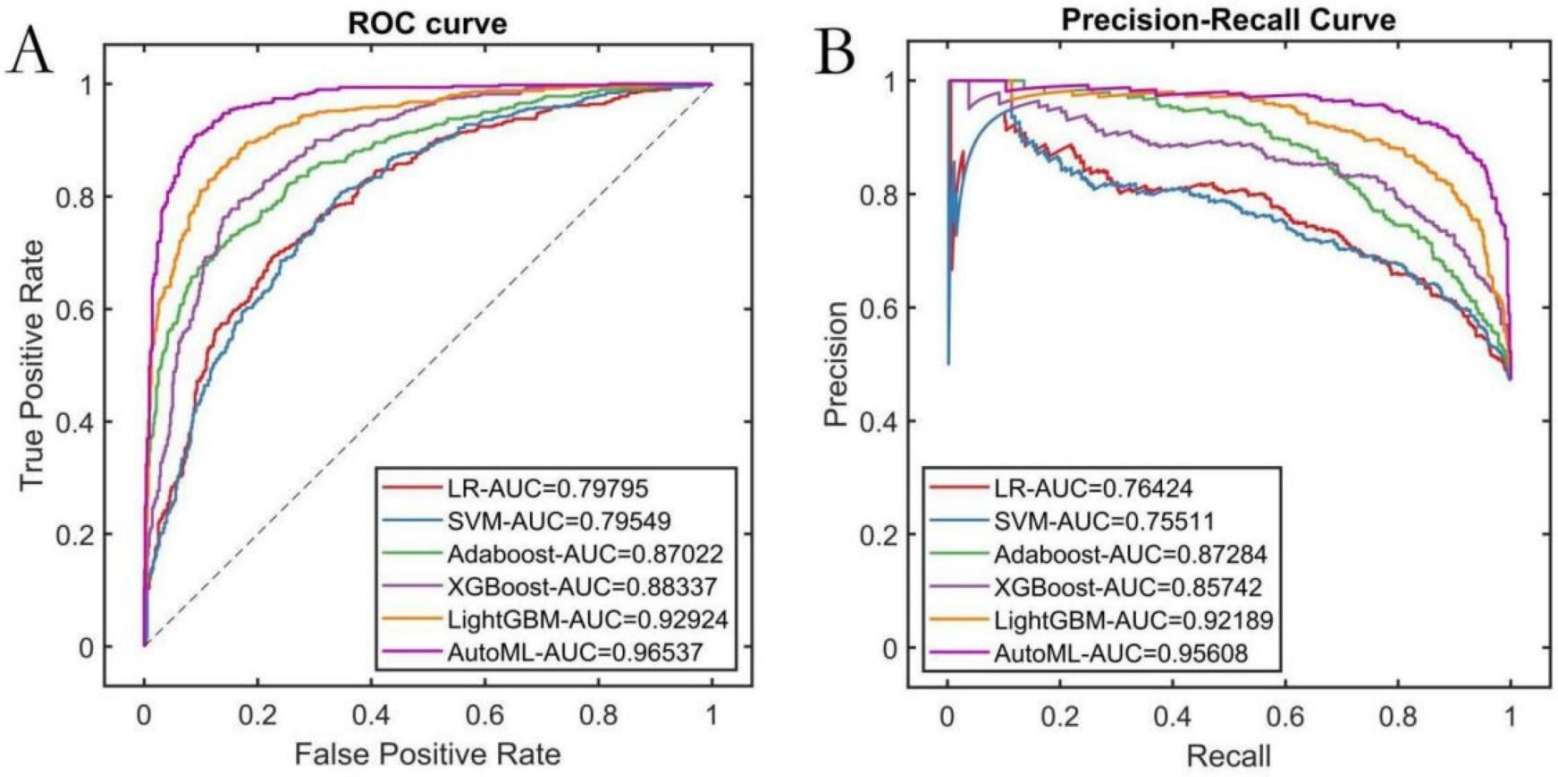
Training set cross-validation performance. **(A)** ROC curve; **(B)** PR curve.

The study systematically evaluated the predictive performance of six ML models on the training set. The AutoML model exhibited optimal overall performance, achieving a ROC-AUC of 0.9654 and PR-AUC of 0.9561. Notably, its F1 score advantage (0.8561) underscored its clinical utility in balancing precision-recall trade-offs. AutoML's key features included: etiology classification, common femoral vein recanalization rate, D-dimer level, stent diameter, presence of arteriovenous fistula, IVC stent length, external iliac vein recanalization rate, CRP level, anticoagulation regimen, APTT, and gender (Table 2; Figure 4).

**Table 2:** Training set cross-validation performance.

Models	PRE	SEN	SPE	ACC	F1	ROC-AUC	PR-AUC
LR	0.4896	0.9900	0.0753	0.5076	0.6552	0.7980	0.7642
SVM	0.6411	0.8540	0.5717	0.7051	0.7324	0.7955	0.7551
Adaboost	0.5814	0.9500	0.3871	0.6531	0.7213	0.8702	0.8728
XGBoost	0.6236	0.9740	0.4731	0.7098	0.7603	0.8834	0.8574
LightGBM	0.6112	0.9840	0.4391	0.6966	0.7540	0.9292	0.9219
AutoML	0.7589	0.9820	0.7204	0.8440	0.8561	0.9654	0.9561

### 3.4 Testing set predictive performance comparison

**Figure 5:**
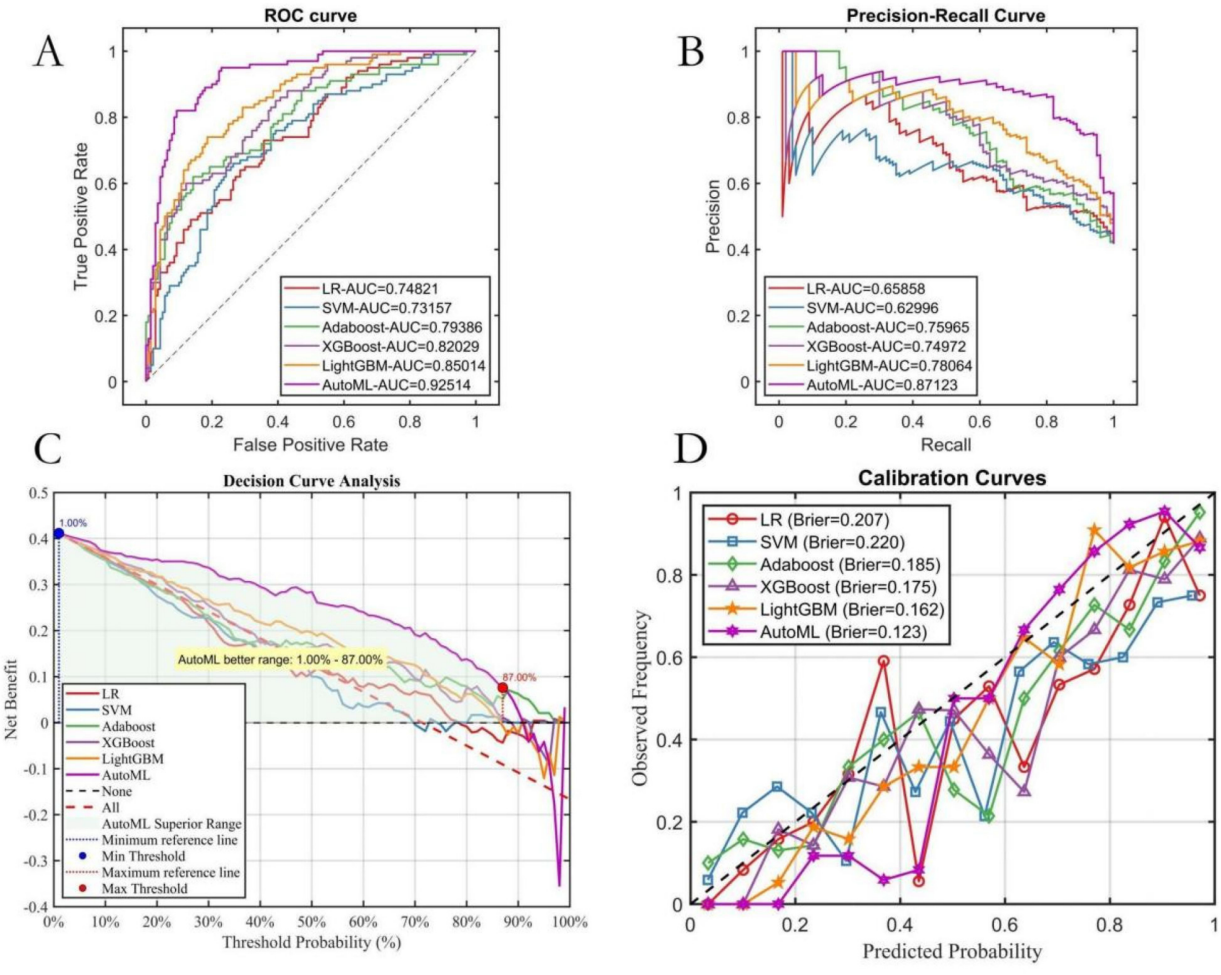
Testing set predictive performance. **(A)** ROC curve; **(B)** PR curve; **(C)** DCA curve; **(D)** Calibration curve.

AutoML demonstrated the strongest robustness in the independent test set, achieving a ROC-AUC of 0.9251 and PR-AUC of 0.8712 (Figures 5A,B). Decision curve analysis (Figure 5C) revealed that AutoML provided greater clinical net benefit than traditional methods at risk thresholds of 1%–87%, maintaining a stable high-level net benefit curve across a broad threshold range, indicating superior generalization. Calibration curve analysis (Figure 5D) confirmed AutoML's optimal calibration (lowest test set Brier score: 0.123). See Table 3 and Figure 5.

**Table 3:** Testing set performance.

Models	PRE	SEN	SPE	ACC	F1	ROC-AUC	PR-AUC
LR	0.4167	1.0000	0.0000	0.4167	0.5882	0.7482	0.6586
SVM	0.5029	0.8800	0.3786	0.5875	0.6400	0.7316	0.6300
Adaboost	0.5923	0.7700	0.6214	0.6833	0.6696	0.7939	0.7597
XGBoost	0.6190	0.7800	0.6571	0.7083	0.6903	0.8203	0.7497
LightGBM	0.5655	0.9500	0.4786	0.6750	0.7090	0.8501	0.7806
AutoML	0.6234	0.9600	0.5857	0.7417	0.7559	0.9251	0.8712

### 3.5 Interpretability analysis

#### 3.5.1 LASSO regression analysis

**Figure 6:**
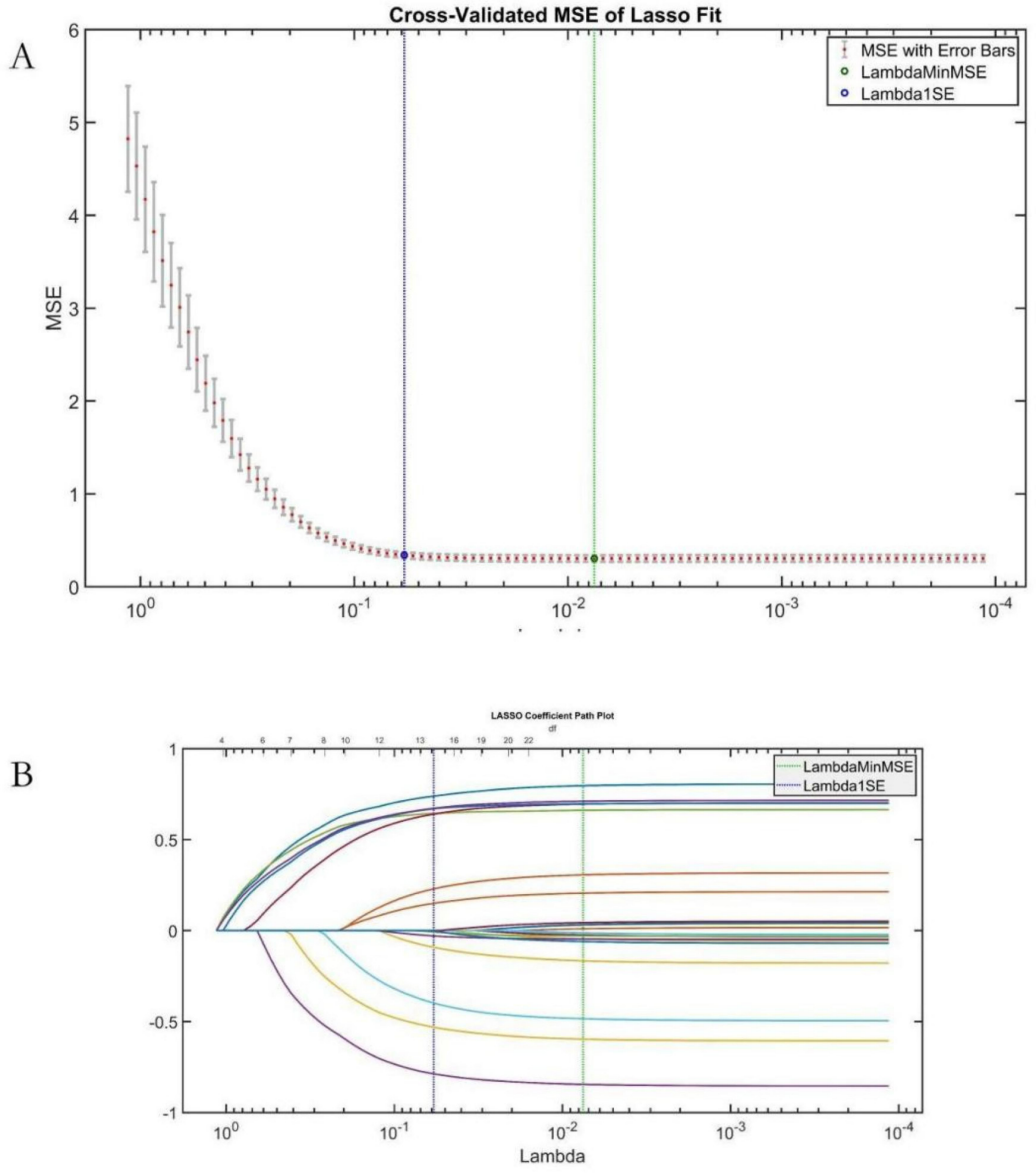
LASSO regression results. **(A)** Coefficient trajectory; **(B)** MSE cross-validation curve.

LASSO regression validated AutoML's feature relevance (Figure 6). The Lambda1 SE criterion selected 14 variables, with a 78.57% overlap (11/14) against AutoML's features: etiology classification, common femoral vein recanalization rate, D-dimer level, stent diameter, arteriovenous fistula, IVC stent length, external iliac vein recanalization rate, CRP, anticoagulation regimen, APTT, gender, surgery duration, FIB, and hospital stay time.

#### 3.5.2 SHAP analysis

**Figure 7:**
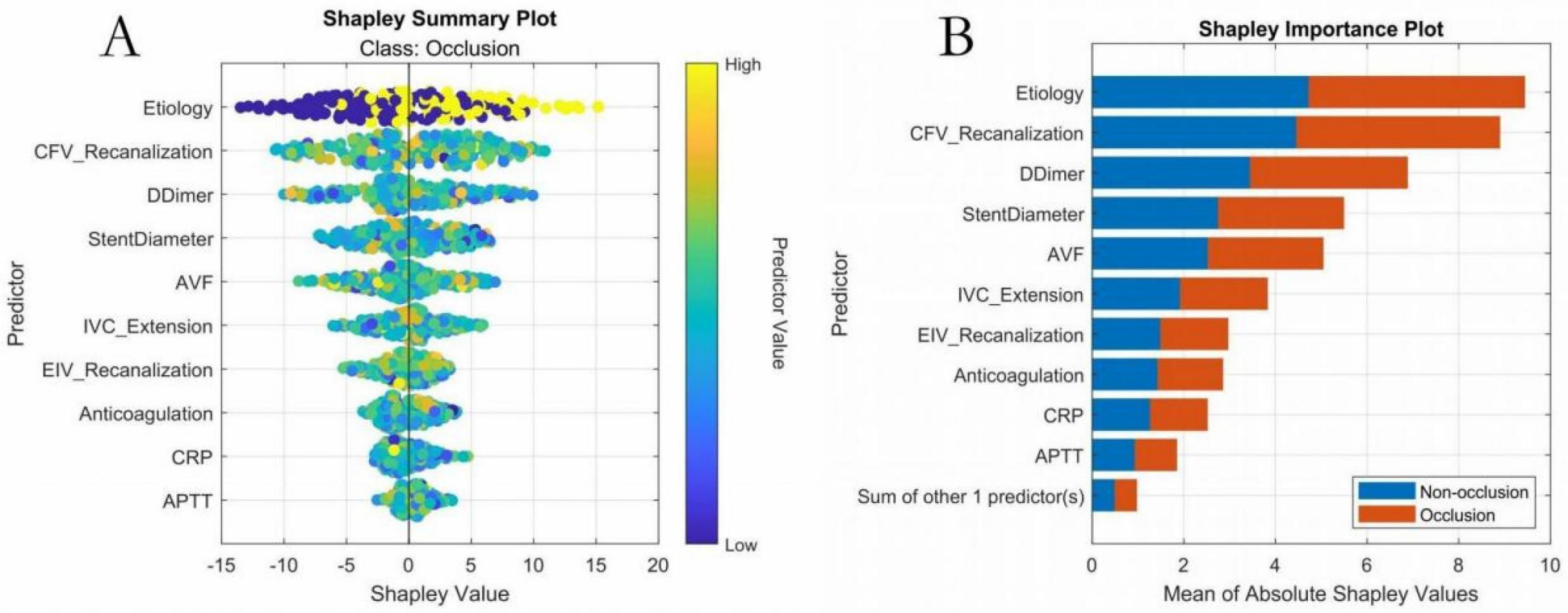
Key features—overall SHAP value comparison. **(A)** SHAP summary plot; **(B)** SHAP feature importance plot.

**Figure 8:**
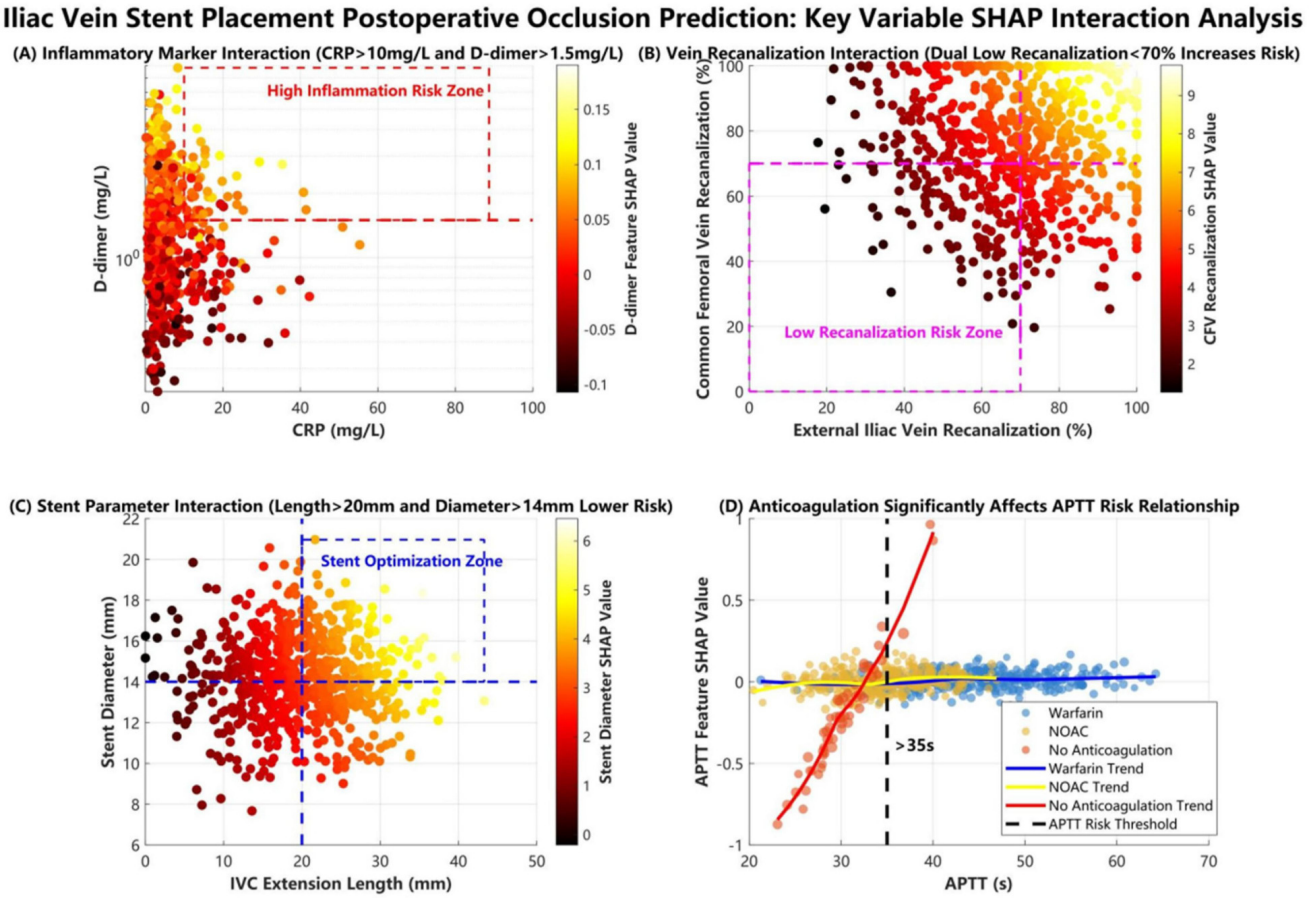
SHAP interaction analysis of key feature pairs.

SHAP ranked key features as follows: (1) etiology classification, (2) common femoral vein recanalization rate, (3) D-dimer level, (4) stent diameter, (5) arteriovenous fistula, (6) IVC stent length, (7) external iliac vein recanalization rate, (8) CRP level, (9) anticoagulation regimen, (10) APTT, (11) gender (Figure 7). Interaction analyses (Figure 8) revealed: (A) Inflammatory synergistic effect: Risk significantly increases when CRP >10 mg/L and D-dimer >1.5 mg/L; (B) Venous recanalization dual insufficiency: Occlusion risk doubled when both common femoral vein and external iliac vein recanalization rates were <70%; (C) Stent parameter optimization: Lower risk occurred with IVC stent length >20 mm and stent diameter >14 mm; (D) Anticoagulation importance: The no-anticoagulation group showed highest risk with prolonged APTT (red line slope indicated significant increase), while anticoagulation therapy reduced risk.

During the SHAP interpretability analysis, we not only visually revealed critical thresholds and interaction effects among key predictors including etiology classification, common femoral vein recanalization rate, and inflammatory-coagulation biomarkers, but also incorporated clinical scenarios to demonstrate translational value. For instance, we identified a nonlinear amplification effect when elevated CRP (>10 mg/L) coexists with high D-dimer levels (>1.5 mg/mL): in such cases, the occlusion probability increases by 32%–45% compared to isolated biomarker elevation. This interaction directly informs clinical management–patients exhibiting this dual-biomarker profile would receive intensified anticoagulation (e.g., switching from single antiplatelet therapy to dual-pathway inhibition) and accelerated imaging surveillance. Another example involves perioperative decision-making: when stent overlap length exceeds 30% of IVC segment length combined with suboptimal recanalization rate (<70%), the model prompts endovascular revision during the index procedure, demonstrating how multidimensional feature interactions guide real-time interventions.

### 3.6 Clinical decision system

**Figure 9:**
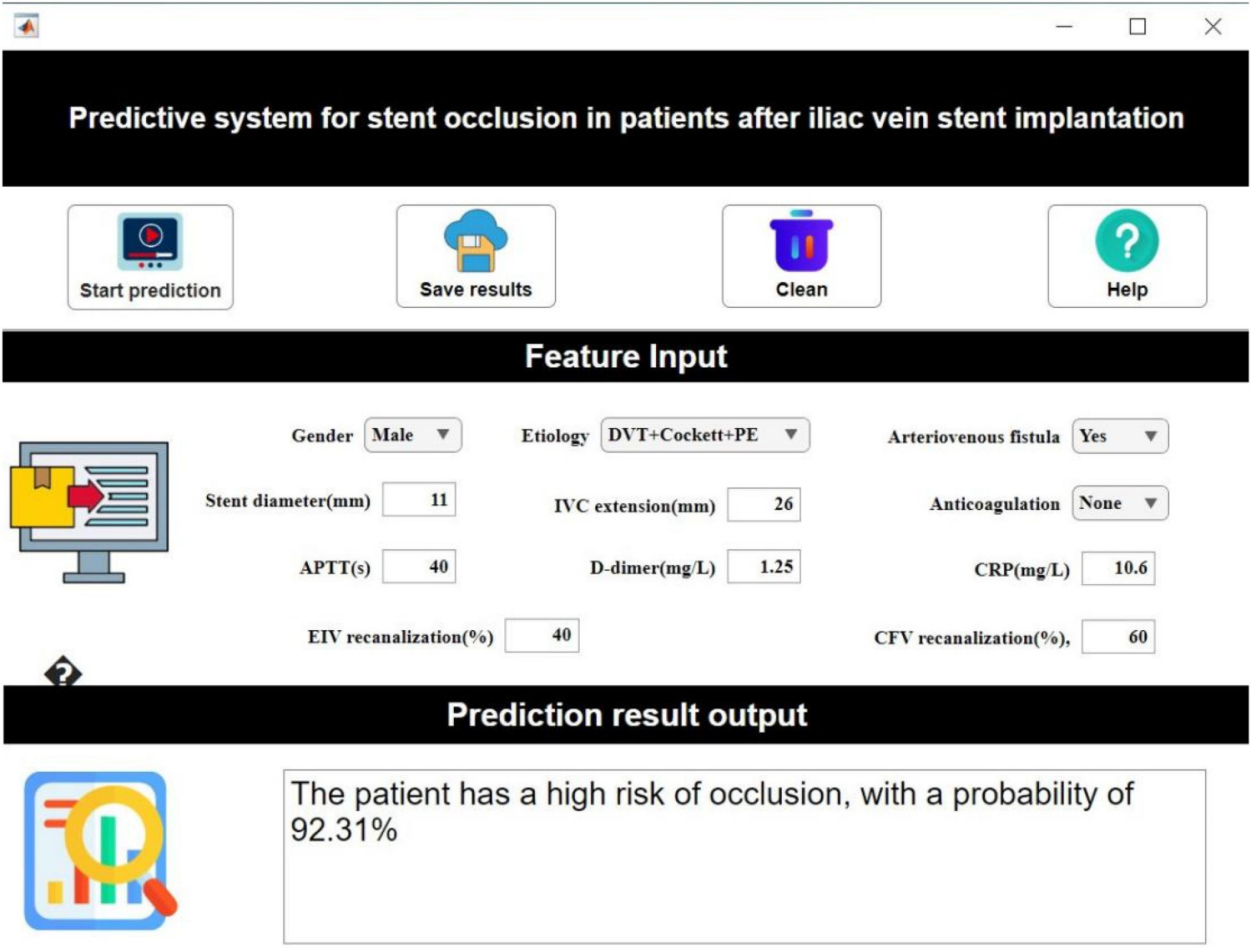
Clinical decision system demonstration.

Based on the established predictive model, our study developed a visualized interactive prediction interface using MATLAB's App Designer. Clinicians input patient key features in the “feature input” panel, after which the system calculates occlusion probability (0%–100%) in real-time based on the trained AutoML model (Figure 9).

## 4 Discussion

Our study provides an in-depth exploration of artificial intelligence applications in the medical field. The most prominent achievement lies in the successful construction and validation of a high-accuracy, high-interpretability predictive model for stent occlusion after iliac vein placement. Specifically: We pioneered an improved Sequoia Optimization Algorithm (ISequoiaOA)-driven AutoML framework for end-to-end modeling. This framework significantly enhances modeling efficiency through automated feature engineering and hyperparameter tuning while maintaining superior discrimination metrics such as ROC-AUC (AUC = 0.903); Innovatively integrating LASSO regression with SHAP interpretability analysis, we visually revealed critical thresholds and interaction effects among key features including etiology classification, common femoral vein recanalization rate, and D-dimer levels for the first time; We developed a MATLAB App Designer-based clinical decision support system that enables real-time visual calculation of occlusion probability. This provides immediate quantitative guidance for anticoagulation regimen adjustment and stent parameter optimization.

Our model profoundly advances venous occlusion prediction by resolving critical limitations in prior frameworks (20–23). Overcoming linear model defects: By identifying nonlinear relationships, we precisely captured the threshold effect where occlusion risk geometrically multiplies when both common femoral vein and external iliac vein recanalization rates fall below 70%—an interaction traditional regression models frequently miss. Dynamic risk assessment: Unlike static prediction tools, we incorporated postoperative dynamic monitoring indicators (e.g., CRP, D-dimer) to enable time-series thrombotic risk evaluation through continuous data updates. Clinical utility validation: Using decision curve analysis, we quantified clinical net benefit, demonstrating that model-assisted decisions yield higher net benefit (NB) than traditional strategies when the threshold probability exceeds 15%, confirming clear clinical translation value. The SHAP-based interpretability pipeline uniquely links nonlinear risk quantifiers to clinical actions—for instance, outputting probability thresholds that trigger targeted interventions like dual-pathway anticoagulation or re-intervention planning. Crucially, this tool holds greatest clinical utility in two complex scenarios: (1) Multifactorial risk escalation—such as patients with acute DVT superimposed on May-Thurner anatomy and CRP >10 mg/L—where the model's interaction detection quantifies synergistic occlusion risk and directly prompts intensified anticoagulation with extended surveillance; (2) Intraoperative decision support for cases of malignant tumor-induced iliac vein compression (e.g., retroperitoneal tumors), where the model's real-time hemodynamic parameter assessment guides the necessity of concomitant IVC filter placement. Such precision management reduces unnecessary interventions in low-risk cohorts while concentrating resources on high-risk patients.

The analysis of correlations between key features and clinical outcomes revealed three core mechanisms. First, the cascade effect of the etiology spectrum was prominent, as SHAP analysis confirmed that the triad etiology of DVT combined with Cockett syndrome and pulmonary embolism (PE) had the highest contribution weight; the mechanism involves the superposition of multiple pathological factors impairing vascular endothelial repair and amplifying coagulation cascades, increasing occlusion risk beyond that in patients with a single etiology, highly consistent with the three-factor theory proposed by previous studies (24–26). Second, the synergistic amplification between hemodynamic and inflammatory indicators was significant, with SHAP interaction plots showing that when CRP levels exceeded 10 mg/L and D-dimer levels exceeded 1.5 mg/L (indicating dual-pathway activation of inflammation and hypercoagulability), the SHAP value sharply increased to 2.8 times the baseline risk; this phenomenon was confirmed at the molecular level, where macrophage infiltration (marked by elevated CRP) upregulates tissue factor expression, forming a positive feedback loop with hypercoagulability that accelerates fibrin deposition on stent surfaces (27–29). Notably, the synergistic effect of postoperative common femoral vein and external iliac vein recanalization rates exhibited threshold characteristics: dual insufficiency (both rates below 70%) induced vortex formation, reducing shear stress and prolonging blood stasis time, triggering a risk-doubling inflection point in SHAP interaction values, providing a basis for targeted hemodynamic interventions (30). Third, stent geometry parameters demonstrated a “double-edged sword” effect, where a diameter exceeding 14 mm reduced metal load density, but an inferior vena cava segment length exceeding 20 mm induced outflow turbulence and damaged venous sinus endothelium.

Although this study has achieved significant outcomes, several limitations warrant consideration. Firstly, the retrospective design inherently limited routine testing of potential predictors like hereditary thrombotic markers, which may underestimate genetic susceptibility. This retrospective approach also introduces possible selection bias due to population heterogeneity, particularly regarding comorbidities and procedural variations across centers. Secondly, stent parameters (e.g., material/weaving method) lacked full standardization due to manufacturer variations, necessitating prospective validation. Thirdly, the clinical decision software developed on MATLAB® limits open reproducibility and requires platform migration for broader implementation. Finally, despite software development, multicenter clinical validation remains pending, demanding randomized trials to assess real-world efficacy. Future research should: conduct prospective multicenter validation (>2,000 cases) while supplementing thrombosis molecular markers (e.g., thrombin-antithrombin complexes, *P*-selectin) to build comprehensive prediction systems; develop temporal models (e.g., LSTM) for real-time risk alerts using postoperative monitoring data; advance feature engineering by integrating ultrasound-derived hemodynamics (e.g., duplex velocities) and computational fluid dynamics (CFD)-based flow indices to optimize stent deployment; and design personalized anticoagulation trials based on model stratification, such as NOAC dosage differentials for risk groups to quantify long-term patency improvements.

In summary, this study successfully constructed an iliac venous stent occlusion prediction system by integrating efficient AutoML modeling with explainable analysis; its core innovation lies in quantifying interactions across multi-level factors and breaking traditional prediction dimension limitations, while validating the practical value of clinical decision support tools for enhancing anticoagulation precision, thereby providing an innovative methodology and evidence-based foundation for refining venous stent protocols and optimizing long-term patient outcomes.

## Data Availability

The raw data supporting the conclusions of this article will be made available by the authors, without undue reservation.
